# A discourse analysis of the macro-structure, metadiscoursal and microdiscoursal features in the abstracts of research articles across multiple science disciplines

**DOI:** 10.1371/journal.pone.0205417

**Published:** 2018-10-12

**Authors:** Sing Bik Cindy Ngai, Rita Gill Singh, Alex Chun Koon

**Affiliations:** 1 Department of Chinese and Bilingual Studies, The Hong Kong Polytechnic University, Hung Hom, Kowloon, Hong Kong SAR, China; 2 Language Centre, Hong Kong Baptist University, Kowloon Tong, Kowloon, Hong Kong SAR, China; 3 School of Life Sciences, Faculty of Science, The Chinese University of Hong Kong, Shatin, N.T., Hong Kong SAR, China; Central European University, HUNGARY

## Abstract

The abstract of a scientific research article convinces readers that the article deserves to be read. Abstracts can also determine the success of publications and grant applications. In recent years, there has been a trend of cross-disciplinary collaborations in the science community. Scientists have been increasingly expected to engage not only experts of their own disciplines, but also other disciplines with the scope of interest extending to non-experts, such as policy-makers and the general public. Thus, the macro-structure, metadiscoursal and microdiscoursal features exhibited in scientific article abstracts merit attention. In our study, we examined 500 abstracts of scientific research articles published in 50 high-impact journals across five science disciplines (Earth, Formal, Life, Physical and Social Sciences), and performed quantitative analysis of the move structure as well as use of boosters and linguistic features. We found significant interdisciplinary variations in the move structure, boosters and linguistic features employed by these science disciplines. We confirmed that each science discipline possesses a distinct set of macro-structural, metadiscoursal and formalization features, which contribute to its own unique discipline-specific convention. Understanding and observing the disciplinary rhetorical choices and communication conventions will allow scientists to align the abstracts of their studies with the expectations of the targeted audience.

## Introduction

An abstract of a scientific research article is defined as “a description or factual summary of the much longer report, and is meant to give the reader an exact and concise knowledge of the full article” [1]. The abstract is the first section of an article, and can be regarded as “a stand-alone genre” allowing readers to identify articles of their interest and determine the article’s relevance [2]. An abstract also facilitates readers in determining whether to read the whole article [3]. While an abstract should summarize, one of its main purposes is to arouse interest and persuade readers that the article deserves to be read [4, 5]. Previous research has confirmed that the abstract and title of a research article can be used to dictate whether the article is worth reading [6, 7]. Furthermore, the scientific article abstract is the only section of the article that is readily available on some online databases and therefore, it offers “…a relatively consistent point of entry to scientific publications” [8](p2). Due to the immense number of academic publications in the globalized science field given that scientists are under immense pressure to publish more output, the abstract plays a more prominent role in orienting readers to select useful articles relevant to their area of expertise [9–11]. The abstract should be newsworthy and attract readers’ attention by highlighting the impact or significance, originality, novelty and professional credibility of the research study while also indicating membership of the academic community of that discipline [4].

In particular, the adoption of rationalization strategies [12] as realized in the move structure (i.e. functional units in the organization of text that dictate the textual structure) [1, 13], and the use of metadiscoursal features such as boosters for persuasion [14] and the linguistic features adopted in formalization strategies [15] have been well-documented. However, a study on how the macro-structure, metadiscoursal and microdiscoursal features vary across multiple science disciplines especially with regards to scientific abstract writing is needed since a study on this has not been undertaken. The increasing competitiveness involved in obtaining grants and publications [16] in the science discipline has meant that abstracts are gaining more importance. Highlighting the interdisciplinary variations evident in abstracts would provide further knowledge of how to construct abstracts to target the intended audience, who may comprise not only experts in that particular discipline but also non-experts and policy-makers.

Rationalization is an important characteristic of academic science discourse in which issues are presented in a logical order such as indicating the cause-effect relations, problem-solution order and arguments for and against different options to enhance reading comprehensibility and save the time of scientists in keeping abreast of the latest research findings [17]. Logical structuring of ideas allows for the realization of rationalization strategies [12, 18, 19]. This logical and cognitive structuring can be realized in the move structure of a discourse, which is a text structure (i.e. Introduction-Method-Results-Discussion-Conclusion) that facilitates understanding of the abstract [4, 20, 21]. For instance, rationalization strategies such as defining a problem and identifying a cause are manifested in the Introduction move while the strategies of presenting outcomes of investigations, arguments to legitimize findings and possible solutions are exhibited in the Results move in the abstract.

The structure of the abstract should align well with the way the article is structured, facilitating readers to predict the content of the upcoming text and ease their understanding. Prior analysis of abstracts has found a rhetorical macrostructure, which is consistent with the standardized structure of the research article: Introduction, Methods, Results and Conclusion [1, 13]. Subsequently, the move structure was examined in 800 abstracts from 10 journals in 1997 across disciplines in linguistics, philosophy, marketing, physics and biology, electrical and mechanical engineering, and sociology by distinguishing the purpose move from the introduction move, and the following five organizational move structure was proposed: (1) Introduction, (2) Purpose, (3) Method, (4) Results and (5) Conclusion (a.k.a. the 5-step move structure of “I-P-M-R-C”) [4].

While our study draws partially on prior research [4], we incorporated the use of rationalization strategies as realized in the move structure and acknowledge that there may be distinct variations in the move structure of abstracts across different science disciplines. In other words, our study is primarily concerned with the possible interdisciplinary variations with regards to how rationalization strategies are manifested in the macro-structure of abstracts.

Apart from macro-structure, abstracts also rely on meta-discourse to negotiate the meanings in texts and engage the audience of a particular community [22]. Interactional meta-discourse focuses on how scientists highlight their claims to inform the audience while increasing credibility and acceptance of such claims [22]. The claiming of novel and credible findings often involves the balanced use of boosters and hedges [14]. When presenting findings, certainty is often required to gain peers’ acceptance of the researcher’s claims/evidence. This is accomplished through the use of boosters, which enable researchers to confirm their findings with certainty, establish novelty and credibility, as well as engage their academic peers and increase solidarity with them [14, 17, 23]. Nevertheless, on certain occasions, tentativeness may be required, through the use of hedges, to acknowledge the possibility that the findings are subject to interpretation and to soften criticism from peers in the academic community [12, 14, 24, 25]. Boosters consist of adverbs and adverbials (e.g. *evidently*, *certainly*), reporting verbs (e.g. *demonstrate*, *indicate*, *show*), certainty modals (*must*, *have to*), attitude stance markers (e.g. *remarkably*, *interestingly*), emphatic adverbs (e.g. *clearly*, *absolutely obviously*), intensifiers (*extremely*, *very*, *crucial*, *essential*), adjectives (*obvious*, *apparent*, *evident*) and verbs such as *‘will’* [4, 17]. Since the abstract of scientific research articles serves as “a promotional genre”, it is likely that boosters instead of hedges would be utilized to highlight novel and original findings. Therefore, concerning the analysis of metadiscoursal features, we focused solely on boosters in our study.

While metadiscoursal features like boosters can underpin rhetorical certainty, other linguistic features can give rise to different writing styles such as formal, conversational and colloquial styles [17, 26]. Quantitative analysis of linguistic features can be performed through examining the sentence length, word length, parts of speech (POS), number of noun-beginning, pronoun-beginning and WH-pronoun beginning sentences [26]. Formalization or formality in science research articles is exhibited in the frequent use of nouns and prepositional phrases at the beginning of sentences and the use of longer sentence structures [17, 23, 26, 27]. On the other hand, a conversational style of writing includes the use of first person pronouns (e.g. “I” and “you’ to address readers), pronoun-beginning and WH-pronoun beginning sentences, contractions, and imperative structures [17, 26–28]. Abstracts also frequently use the third person and passive voice [20]. The use of passive voice distances the writer from the discipline, placing more emphasis on the topic than the writer [17], thus strengthening the perception of science as an impersonal discipline.

Whereas formalization aims at reducing ambiguity and misinterpretation of information, conveys impartiality, precision and objectivity, and minimizes the individual interests of researchers [15], its excessive use may distance non-experts from the abstract. In contrast, informality can be employed by researchers to show a willingness to negotiate claims of knowledge and to acknowledge subjectivity of some claims [15]. Since there is a dearth of literature on how formalization is used in science abstracts, we filled this gap by drawing on previous research studies’ [17, 26] quantitative analyses of linguistic features that manifested formalization in texts, whereby we mainly examined such formalization features exhibited in high-impact abstracts across multiple science disciplines and comparing the interdisciplinary variations in a contemporary context.

In the past, the audience of scientific research articles primarily consisted of scientists or experts familiar with that particular discipline [29]. Thus, fewer rationalization strategies actualized in the move structure and the use of more hedges and formalization were often employed in abstracts intended for the targeted community of researchers. In contemporary times, science communication has experienced a drastic change from a deficit model to a democratic one whereby its audience not only includes scientists from that particular discipline but also non-experts and policy-makers [29, 30]. The presentation of novel findings has gained more importance, and ease of reading and accessibility rely on how the text is structured, how the authority of the researcher is established, and the avoidance of excessive formalization of language to inform the audience of key research ideas.

## Purpose of study and research questions

Prior research on scientific article abstracts has merely focused on one or two aspects such as the rhetorical use of topics in science abstracts [31] and the move structure and use of boosters [4, 14], while our study offers a detailed analysis of a number of aspects exhibited in abstracts as stated above across diverse science disciplines. Since there is limited research on the comprehensive analysis of the science discourse including the move structure, metadiscoursal and microdiscoursal features realized in the rationalization and formalization strategies employed by scientific research article abstracts across multiple science disciplines, we conducted this quantitative study to fill this gap, and in particular, to highlight the interdisciplinary variations with respect to the discourse analysis of abstracts.

We scrutinized the five-step move structure of abstracts across five science disciplines to reveal their genre-specific macro-structural characteristics. Specifically, we examined whether our adapted I-P-M-R-C rhetorical macro-structure manifested rationalization strategies as observed in 500 abstracts of the top 50 journals from five major science disciplines. In addition, we investigated these abstracts with a view to determining the frequency of metadiscoursal features, boosters in particular, and the extent of formalization in terms of longer sentence length, word length (e.g. complex multi-level words), frequent use of nouns, noun-beginning sentences and object-verb constructions with a less frequent use of pronouns and WH-pronouns at the beginning of sentences. We focused on these features since identifying them is intricately linked to the rhetorical context in which they operate and the scientist’s intent. These rhetorical choices in science abstracts need to be understood in the current, academic institutional context in which competition for grants has increased and the need to publish has soared.

Therefore, the following research questions were investigated:

RQ1: Was the five-step move structure of I-P-M-R-C exhibited in the rationalization strategies commonly adopted in the scientific article abstracts across the five science disciplines? Were there significant variations between the move structures of abstracts in different science disciplines?

RQ2: Were there variations in the use of metadiscoursal features (i.e. boosters) in the abstracts across different science disciplines?

RQ3: To what extent were formalization features exhibited in the science abstracts across different science disciplines? Were there differences in how these features were employed across the disciplines?

## Materials and methods

Our study examined the science research article abstracts across five science disciplines by focusing on the rhetorical move structure for rationalization, metadiscoursal features such as the use of boosters, and linguistic features adopted in the formalization strategy in the sampled 500 abstracts. Both computational method and qualitative content analysis were employed in this study to provide answers to the research questions.

### Sampling method

To facilitate the investigation of textual features employed in the science research article abstracts, we selected abstracts from high-impact science research journals from five major disciplines. These disciplines included Earth, Formal, Life, Physical and Social Sciences, as well as their sub-disciplines including Geology and Geosciences in Earth Sciences, Mathematics and Computer Sciences from Formal Sciences, Biology and Medicine from Life Sciences, Chemistry and Physics from Physical Sciences and Sociology and Psychology from Social Sciences.

Fifty journals of the highest-impact factor and total citations from the sub-disciplines of the five major science disciplines were identified from InCites Journal Citation Report 2016 (see Supporting Information–S1 File for the science journals shortlisted). To avoid content and structural bias, journals that had a standardized abstract format and/or focused on only publishing review articles were not selected even if they had a high-impact factor.

Due to the enormous number of abstracts published and the imbalance in the number of abstracts from different disciplines, we adopted the Sample Size Calculator constructed and operated by the National Statistical Service (n.d.) at the Australia Bureau of Statistics for the calculation of the sample size required. We sampled 500 abstracts (confidence level at 99% and confidence interval at 0.0576) from the top 50 journals (i.e. 10 abstracts per journal) in the above disciplines for the compilation of our database (102,048 words) for comparison. These abstracts were collected using a systematic random sampling method. For instance, if Journal A published 520 articles with abstracts in the year of 2015 to 2016, we selected one abstract from every set of 52 articles to harvest the required 10 abstracts.

### Coding scheme and procedure

To develop the coding scheme for content analysis of the move structure [32], use of boosters and linguistic features employed in the sampled abstracts, we drew on previous literature [4, 14, 17, 26, 32]. We studied the constructs used for examining the strategies in the empirical database. For RQ1, we coded the steps in all 500 sampled abstracts based on the I-P-M-R-C structure for a scientific research article abstract:

1. Introduction: Set the context of the article by providing background information on the paper, the cause or problem or need for the research, or the significance of the research.
2. Purpose: State the purpose/aim, thesis or hypothesis and give a brief outline of the aim of the paper.
3. Method: Give details about the research design, sample, approach, procedure and data.
4. Results: Provide the main findings or the arguments or the solutions or the effects or what was achieved.
5. Conclusion: Interpret the findings of the paper, draw inferences, apply/generalize the findings, and provide the wider implications and significance/value of the research to the discipline.

When coding the steps, we specifically looked for (a) the number of step(s) employed, (b) the missing step(s), (c) any additional step(s) uncovered (including but not limited to theme issues in current studies, author(s) information, journal information and funding schemes) and (d) any reverse sequence of steps. To further investigate the sequence of the steps, we coded the initial step(s) of each abstract. Although the use of algorithm and statistical machine learning can help examine the rhetorical functions of topics at the sentence level in a large textual dataset [31, 33], manual coding could ensure a close reading for text analysis [34]. As suggested in a prior study, the use of statistical measures needs to be integrated with a qualitative analysis [35]. In our study, coding within sentences and between sentences was allowed to examine the use of steps more thoroughly. We also coded and surveyed the content delivered in the additional steps and the sequence of steps.

For RQ2, we examined the use of boosters based on the list of boosters suggested in a prior study [4]. We adapted the procedures from corpus-based content analysis, a qualitative-quantitative inference-making methodology for semantic analysis [36]. This method allowed us to make use of “tools from corpus linguistics and computational linguistics to identify term variants and word sense” [36](p9), which in turn allowed us to conduct text-mining of items from the search word lists and quantitative analysis of the mined occurrences [36]. The syntagmatic semantic clustering can be identified with the help of exhibit of word lists or concordance analyses provided by the corpus linguistics software, WordSmith [37], which is a computational tool specifically designed for lexical and semantic analysis in a large database [36, 38]. We therefore employed the *Wordlists* function of WordSmith 6.0 to uncover the frequency of occurrence of 53 boosters suggested in a prior study [4] in the 500 abstracts. Then, we used the *Concordance* in WordSmith to display the occurrence of the top 20 booster(s) and the words co-occurring with the search boosters in the abstracts to examine the location of the boosters in the move structure proposed in RQ1 and reveal the variation in the use of boosters across the five science disciplines.

As corroborated by previous studies, the degree of formality in writing can be determined based on a quantitative analysis of linguistic elements [17, 26]. Therefore, for RQ3, to reveal the use of the formalization strategy across the five science disciplines, we tracked the microdiscoursal features including the number of words, average word length, number of sentences, minimum sentence length, maximum sentence length, average sentence length, object-verb construction, the use of POS (parts of speech) and sentence-initial POS, including the number of nouns, number of noun-beginning sentences, number of pronouns, number of pronoun-beginning sentences and number of WH-pronouns in 500 abstracts using POS tagging tools developed based on Stanford University Part-Of-Speech-Tagger and multilingual text mining tools made available on internet.

Three coders comprising of one research associate and two graduates were comprehensively trained to conduct the coding of the rhetorical move structure, tracking of boosters and linguistic features using computational methods. To ensure inter-coder reliability of the coding of various variables, coders were repeatedly trained on the coding scheme. The measure of inter-coder reliability was based on the co-coding of 10% of the abstracts. This generated acceptable reliability coefficients. For all items coded, the average pairwise percent agreement was greater than 86% and Cohen’s Kappa was greater than 0.804.

### Statistical analysis

One-way analysis of variance (ANOVA) and post hoc Tukey test were employed to generate the results for the research questions regarding the differences in the use of move structure, boosters, and formalization features such as sentence length, word length, the use of POS and sentence-initial POS across the five science disciplines. * *P*<0.05, ** *P*<0.01, *** *P*<0.001, **** *P*<0.0001. All histograms depict mean ± S.E.M.

## Results

### The 5-step move structure was not commonly employed in scientific article abstracts

**Fig 1:**
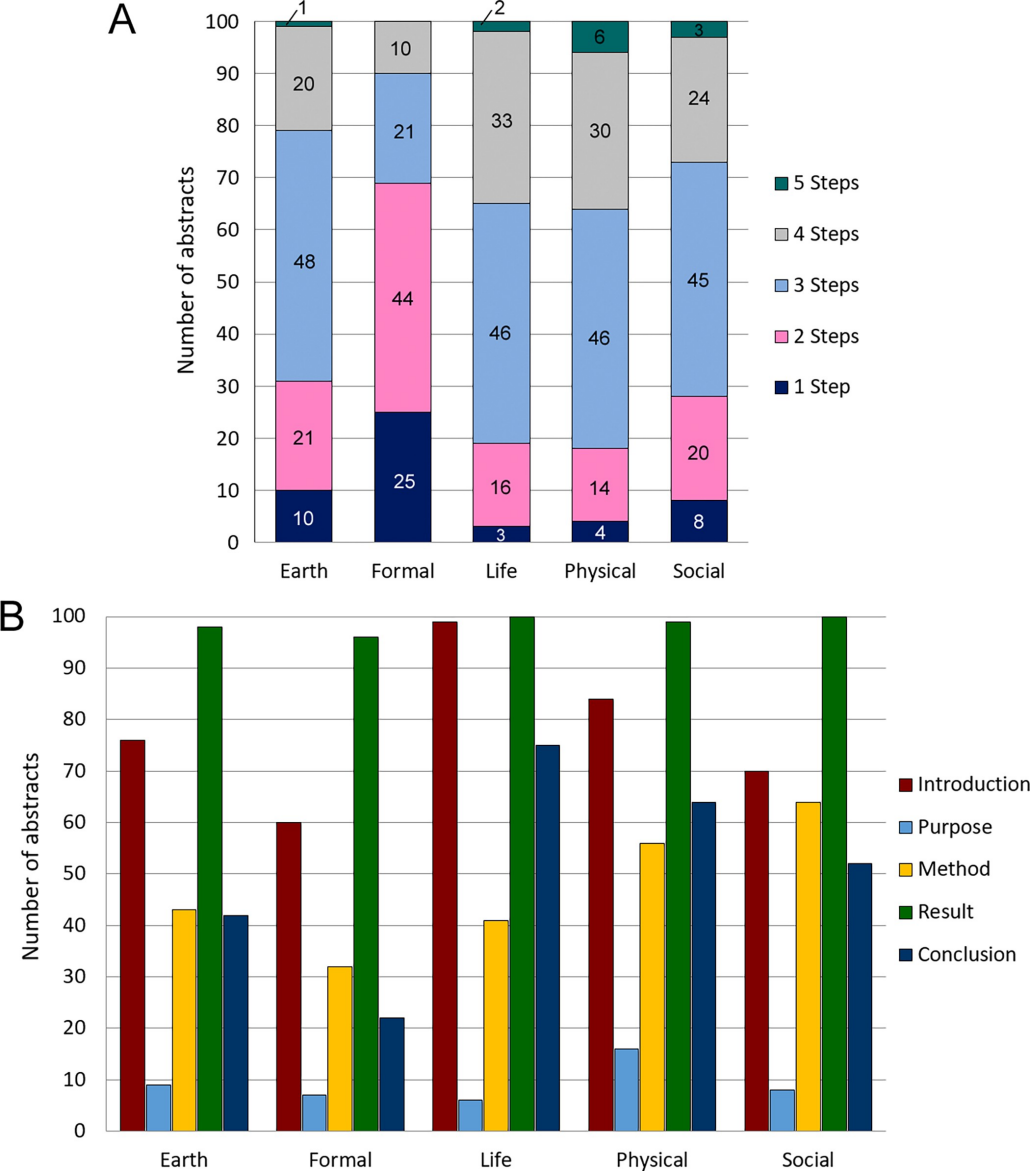
Analysis of the number of steps and distribution of steps in the move structure of science abstracts across various science disciplines. (A) Number of abstracts (out of 100) that contain 1, 2, 3, 4 or 5 steps in each science discipline. Percentage of abstracts (out of all 500 abstracts) containing all 5 steps: 2.4% (12 out of 500); 4 steps: 23.4% (117 out of 500); 3 steps: 41.2% (206 out of 500); 2 steps: 23% (115 out of 500); and 1 step: 10% (50 out of 500). (B) Distribution of steps found in academic abstracts across various science disciplines. n = 100 per science discipline. Each n represents one abstract.

To investigate whether the move structure of I-P-M-R-C [4] is generally employed in scientific article abstracts (RQ1), we quantified the number of steps in 100 science abstracts in each of the five science disciplines (Earth, Formal, Life, Physical and Social Sciences). We observed diverse, salient variations in the move structure of abstracts from different science disciplines. Overall, out of the total 500 abstracts, only 12 abstracts (1 from Earth, 2 from Life, 6 from Physical and 3 from Social Sciences) employed the full 5-step structure, which accounted for only 2.4% of all the abstracts analyzed (Fig 1A). A 4-step move structure was adopted in 23.4% of the abstracts (117 out of 500); a 3-step structure was employed in 41.2% of the abstracts (206 out of 500); a 2-step structure defined 23% of the abstracts (115 out of 500); and a 1-step structure was presented in 10% of the abstracts (50 out of 500) (Fig 1A). Out of the 12 abstracts with all five steps, half of them were from the Physical Science discipline. Remarkably, the 5-step approach was completely absent from the Formal Science discipline (Fig 1A). Most disciplines, including Earth, Life, Physical and Social Sciences, favored the use of a 3-step move structure (41%) while a 1- and 2-step move structures were particularly common in the Formal Science discipline (Fig 1A).

Detailed analysis of the distribution of steps in the move structures across the five science disciplines revealed that Step 4- the presentation of Results (493 out of 500) was the most commonly observed step, followed by Step 1- the Introduction (389 out of 500), Step 5- the Conclusion reached (255 out of 500), Step 3- the Method of investigation (236 out of 500) and Step 2- the Purpose of the research (46 out of 500) (Fig 1B). In general, abstracts of Formal Science research papers accounted for the lowest number for any given step (Fig 1B). On the other hand, 99 out of 100 abstracts in Life Science papers contained Step 1- Introduction and all 100 abstracts included Step 4- Results (Fig 1B), reflecting the importance of these steps in the abstracts of Life Sciences.

### Diverse variations in the rhetorical move structure across multiple science disciplines

**Fig 2:**
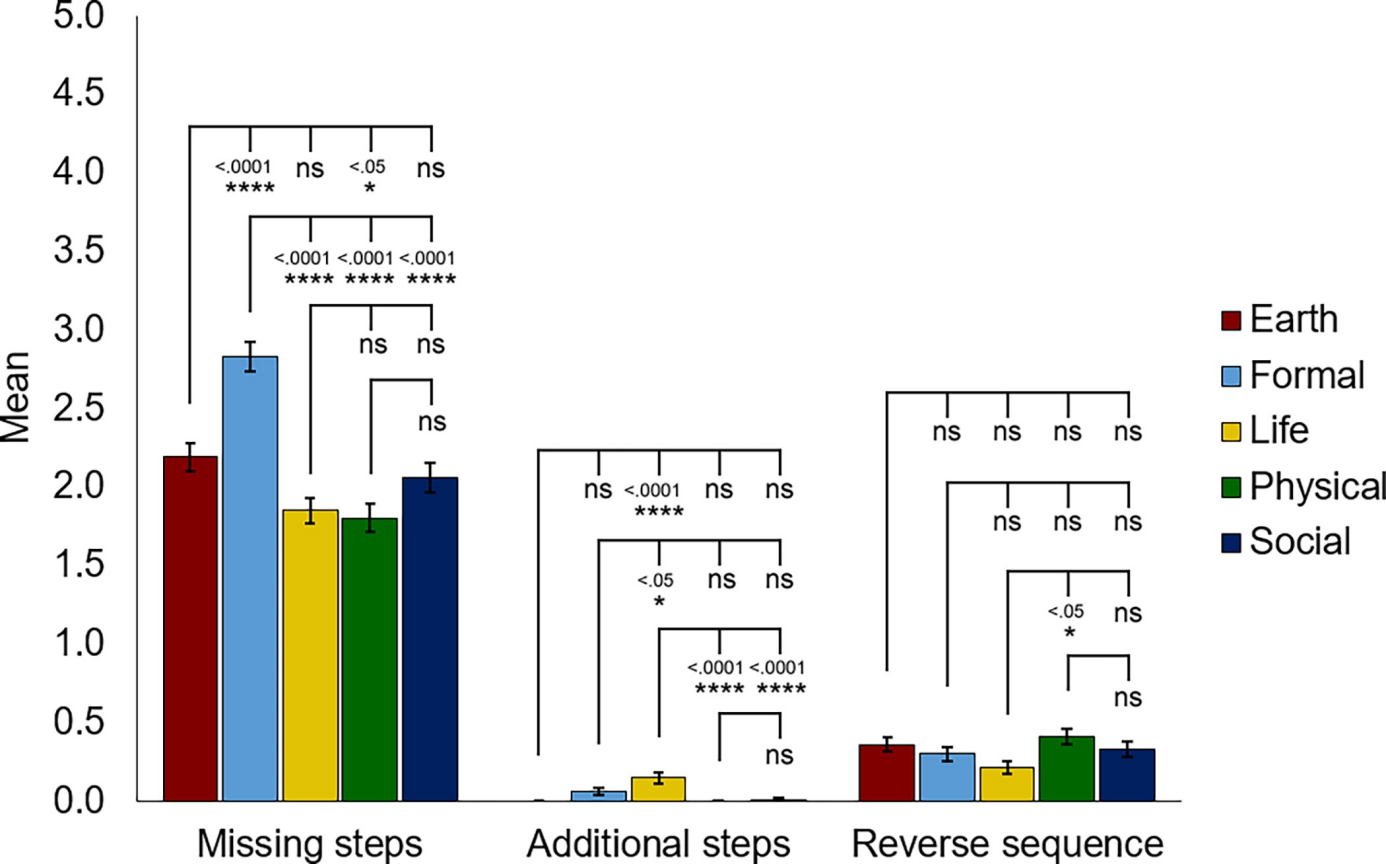
Statistical analysis of rhetorical move structure variations across five science disciplines. Analysis of the number of missing steps, additional steps and reverse sequence in abstracts across various science disciplines. Statistical analysis was performed using ANOVA with post hoc Tukey test. Error bars represent S.E.M. n = 100 per science discipline. Each n represents one abstract.

To further examine if there were significant differences between the move structures of abstracts from the range of different science disciplines, we employed one-way ANOVA and post hoc Tukey test to cross compare the number of missing steps, additional steps (steps that do not belong to any of the five common steps) and reverse sequence (steps not arranged in the common order of I-P-M-R-C) revealed in the scientific article abstracts from different disciplines. Significant differences were found in all three categories: missing steps (F = 21.645, p<0.0001****), additional steps (F = 10.549, p<0.0001****) and reverse sequence (F = 2.578, p<0.05*). Regarding the number of missing steps in the move structure, significantly more missing steps were noted in Formal Science abstracts than those of all the other disciplines (Fig 2). In contrast, a significantly higher number of additional steps were observed in Life Science abstracts than those of all the other disciplines (Fig 2). In other words, there was a greater tendency to employ a simpler move structure in Formal Science abstracts, whereas the propensity to introduce additional content elements in Life Science abstracts was higher. Most additional step(s) found in Life Science abstracts pertained to theme issues in current studies, author(s) information, journal information and funding schemes. Regarding reverse sequences, the Physical Science discipline demonstrated a slightly although significantly higher rate of occurrence than that found in the Life Science discipline.

In probing the frequency of initial step(s) employed in the move structure, we identified and tested 14 combinations of initial steps in abstracts across the science disciplines. Overall, we found that the I-R initiate structure was significantly more common than the I-P initiate (M_I-R_ 0.53 vs M_I-P_ 0.04, p<0.0001****) and the I-M initiate (M_I-R_ 0.53 vs M_I-M_ 0.17, p<0.0001****) structure across all disciplines (Table 1). Table 1 shows the mean frequency and standard deviation of the initial step(s) in the move structure across various science disciplines, while Table 2 shows the Post hoc Tukey test results. Our findings suggested that Formal Science academics employed the I-M-R initiate structure less frequently than those working in Life, Physical and Social Science disciplines (Tables 1 and 2). By contrast, the number of Life Science abstracts with I-R-C initiate structures was significantly larger than that discerned in abstracts from all the other disciplines (Tables 1 and 2). For simpler move structures involving only 1 or 2 steps, Formal Sciences employed significantly more I-R-only and R-only structures than those deemed appropriate in Life, Physical and Social Science disciplines (Tables 1 and 2).

**Table 1:** Frequency of different initial step(s) in the move structure across various science disciplines.

	Move sequence	Science Disciplines									
		Earth		Formal		Life		Physical		Social	
		M	SD	M	SD	M	SD	M	SD	M	SD
I-M Initiate	1. I-M-P initiate	0.01	0.10	0.00	0.00	0.00	0.00	0.04	0.20	0.00	0.00
	2. I-M-R initiate	0.13	0.34	**0.01**	0.10	0.25	0.44	0.16	0.37	0.26	0.44
I-P initiate	3. I-P-M initiate	0.00	0.00	0.01	0.10	0.02	0.14	0.04	0.20	0.04	0.20
	4. I-P-R initiate	0.02	0.14	0.02	0.14	0.02	0.14	0.01	0.10	0.01	0.10
I-R Initiate	5. I-R	0.13	0.34	**0.24**	0.43	0.10	0.30	0.10	0.30	0.07	0.26
	6. I-R-C initiate	0.25	0.44	0.10	0.30	**0.41**	0.49	0.25	0.44	0.16	0.37
	7. I-R-I initiate	0.01	0.10	0.00	0.00	0.02	0.14	0.03	0.17	0.00	0.00
	8. I-R-M initiate	0.17	0.38	0.15	0.36	0.09	0.29	0.18	0.39	0.15	0.36
	9. I-R-P initiate	0.01	0.10	0.00	0.00	0.01	0.10	0.02	0.14	0.00	0.00
R initiate	10. R	0.11	0.31	**0.22**	0.42	0.02	0.14	0.04	0.20	0.08	0.27
	11. R-M initiate	0.06	0.24	0.11	0.31	0.00	0.00	0.06	0.24	0.10	0.30
	12. R others	0.06	0.24	0.08	0.27	0.03	0.17	0.04	0.20	0.04	0.20
	13. M-R initiate	0.01	0.10	0.02	0.14	0.00	0.00	0.01	0.10	0.06	0.24
	14. Others	0.03	0.17	0.04	0.20	0.03	0.17	0.02	0.14	0.03	0.17

“M” stands for mean and “SD” stands for standard deviation. Bolded and underlined text denotes the mean frequency of a particular move sequence under a discipline that is significantly higher or lower than at least three other disciplines based on the post hoc test results in Table 2. n = 100 per science discipline. Each n represents one abstract.

**Table 2:** Post hoc Tukey test results of the frequency of initial step(s) in the move structure across various science disciplines.

	Move sequence	Multiple Comparisons (Post hoc Tukey test results)									
		EF	EL	EP	ES	FL	FP	FS	LP	LS	PS
		P value									
I-M Initiate	1. I-M-P initiate	ns	ns	ns	ns	ns	< .05 *	ns	< .05 *	ns	< .05 *
	2. I-M-R initiate	ns	ns	ns	ns	< .0001 ****	< .05 *	< .0001 ****	ns	ns	ns
I-P initiate	3. I-P-M initiate	ns	ns	ns	ns	ns	ns	ns	ns	ns	ns
	4. I-P-R initiate	ns	ns	ns	ns	ns	ns	ns	ns	ns	ns
I-R Initiate	5. I-R	ns	ns	ns	ns	< .05 *	< .05 *	< .01 **	ns	ns	ns
	6. I-R-C initiate	ns	< .05 *	ns	ns	< .0001 ****	ns	ns	< .05 *	< .001 ***	ns
	7. I-R-I initiate	ns	ns	ns	ns	ns	ns	ns	ns	ns	ns
	8. I-R-M initiate	ns	ns	ns	ns	ns	ns	ns	ns	ns	ns
	9. I-R-P initiate	ns	ns	ns	ns	ns	ns	ns	ns	ns	ns
R initiate	10. R	ns	ns	ns	ns	< .0001 ****	< .0001 ****	< .01 **	ns	ns	ns
	11. R-M initiate	ns	ns	ns	ns	< .05 *	ns	ns	ns	< .05 *	ns
	12. R others	ns	ns	ns	ns	ns	ns	ns	ns	ns	ns
	13. M-R initiate	ns	ns	ns	ns	ns	ns	ns	ns	< .05 *	ns
	14. Others	ns	ns	ns	ns	ns	ns	ns	ns	ns	ns

EF: Earth-to-Formal comparison; EL: Earth-to-Life comparison; EP: Earth-to-Physical comparison; ES: Earth-to-Social comparison; FP: Formal-to-Physical comparison; FS: Formal-to-Social comparison; LP: Life-to-Physical comparison; LS: Life-to-Social comparison; PS: Physical-to-Social comparison.

* *P*<0.05

** *P*<0.01

*** *P*<0.001

**** *P*<0.0001.

n = 100 per science discipline. Each n represents one abstract.

### Use of boosters across the science disciplines

To investigate whether there are variations in the use of metadiscoursal features in the abstracts across different science disciplines (RQ2), we recorded the use of boosters in all 500 abstracts. We first examined the frequency of boosters used based on the list of boosters suggested in a prior study [4]. Table 3 shows the 53 boosters and their frequency of use in the abstracts across the different science disciplines. The most frequently employed boosters, including “show”, “find”, “demonstrate”, “determine”, and “know/known”, were found in all science disciplines (Table 3). We found that Life Science abstracts used more boosters than other disciplines, whereas Physical Science abstracts used the lowest number of boosters (Table 3).

**Table 3:** 53 boosters and their frequency of use across the science disciplines.

Boosters	Frequency of use in total	Earth	Formal	Life	Physical	Social
show	283	52	58	78	58	37
find	116	15	7	48	15	31
demonstrate	108	10	16	34	34	14
determine	60	23	6	14	7	10
know/ known	57	13	15	15	8	6
evidence	53	11	4	13	4	21
prove	40	1	33	1	5	0
essential	25	5	2	12	4	2
given	22	4	11	2	0	5
particularly	20	2	5	6	1	6
certain/ certainly/ certainty	19	3	6	2	3	5
confirm	19	4	0	8	4	3
clear/ clearly	16	5	1	5	3	2
conclude/ conclusive/ conclusively	13	3	0	3	1	6
more than	13	4	1	4	2	2
think	13	2	0	0	1	10
expect	12	4	1	4	1	2
indeed	10	1	1	4	2	2
must	10	1	3	2	0	4
precise/ precisely	8	3	1	2	1	1
always	7	2	1	1	2	1
manifest/ manifestly	6	0	1	1	2	2
never	6	1	0	3	0	2
reliable/ reliably	5	1	1	1	1	1
well-known	5	1	3	0	0	1
true	4	1	3	0	0	0
actually	2	1	0	0	0	1
convince/ convincingly	2	0	1	0	0	1
inevitable/ inevitably	2	1	0	1	0	0
necessarily	2	1	1	0	0	0
obvious/ obviously	2	0	0	0	1	1
assured	1	0	0	0	0	1
definite/ definitely	1	0	1	0	0	0
in fact	1	1	0	0	0	0
unambiguous/ unambiguously	1	0	0	0	1	0
undoubted/ undoubtedly	1	1	0	0	0	0
of course	0	0	0	0	0	0
decided/ decidedly	0	0	0	0	0	0
doubtless	0	0	0	0	0	0
the fact that	0	0	0	0	0	0
impossible/ impossibly	0	0	0	0	0	0
improbable/ improbably	0	0	0	0	0	0
no doubt	0	0	0	0	0	0
patently	0	0	0	0	0	0
perceive	0	0	0	0	0	0
sure/ surely	0	0	0	0	0	0
surmise	0	0	0	0	0	0
unarguably	0	0	0	0	0	0
undeniable/ undeniably	0	0	0	0	0	0
unequivocal/ unequivocally	0	0	0	0	0	0
unmistakable/ unmistakably	0	0	0	0	0	0
unquestionable/ unquestionably	0	0	0	0	0	0
wrong/ wrongly	0	0	0	0	0	0
Total	965	177	183	**264**	*161*	180

Bolded and underlined text denotes the overall highest use of boosters by Life Sciences. Italicized and underlined text denotes the lowest use of boosters by Physical Sciences.

To further examine the use of different boosters across the science disciplines using statistical analyses, especially the frequently used boosters, we performed ANOVA and post hoc Tukey test on the use of the top 10 boosters across the disciplines. We found that Life Science abstracts used boosters such as “show” and “find” significantly more often than the other four science disciplines (Tables 4 and 5), whereas both Life and Physical Science disciplines used “demonstrate” significantly more often than the other three disciplines (Tables 4 and 5). Earth Sciences used “determine” more frequently as opposed to Formal, Physical and Social Science disciplines (Tables 4 and 5). “Prove” in Formal Science abstracts was opted for on a more significant basis compared to writing in the other four science disciplines whereas “given” was also preferred by writers in Formal Sciences than those from Life and Physical Sciences (Tables 4 and 5). Furthermore, Social Sciences utilized the booster “evidence” more often than their Formal and Physical Science counterparts (Tables 4 and 5).

**Table 4:** Descriptive statistics of the top 10 boosters employed across various science disciplines.

	Science Disciplines									
	Earth		Formal		Life		Physical		Social	
Booster	M	SD	M	SD	M	SD	M	SD	M	SD
1. Show	0.52	0.50	0.58	0.50	**0.78**	0.42	0.58	0.50	0.37	0.49
2. Find	0.15	0.36	0.07	0.26	**0.48**	0.50	0.15	0.36	0.31	0.46
3. Demonstrate	0.10	0.30	0.16	0.37	**0.34**	0.48	**0.34**	0.48	0.14	0.35
4. Determine	**0.23**	0.42	0.06	0.24	0.14	0.35	0.07	0.26	0.10	0.30
5. Know/Known	0.13	0.34	0.15	0.36	0.15	0.36	0.08	0.27	0.06	0.24
6. Evidence	0.11	0.32	0.04	0.20	0.13	0.34	0.04	0.20	**0.21**	0.41
7. Prove	0.01	0.10	**0.33**	0.47	0.01	0.10	0.05	0.22	0.00	0.00
8. Essential	0.05	0.22	0.02	0.14	0.12	0.33	0.04	0.20	0.02	0.14
9. Given	0.04	0.20	**0.11**	0.31	0.02	0.14	0.00	0.00	0.05	0.22
10. Particularly	0.02	0.14	0.05	0.22	0.06	0.24	0.01	0.10	0.06	0.24

“M” stands for mean and “SD” stands for standard deviation. Bolded and underlined text denotes the mean of a particular discipline that is significantly higher than at least 3 other disciplines based on the post hoc Tukey test results in Table 5. n = 100 per science discipline. Each n represents one abstract.

**Table 5:** Post Hoc Test results of the Top 10 boosters employed across various science disciplines.

	Multiple Comparisons (Post hoc Tukey test results)									
Booster	EF	EL	EP	ES	FL	FP	FS	LP	LS	PS
	P value									
1. Show	ns	< .01 **	ns	ns	< .05 *	ns	< .05 *	< .05 *	< .0001 ****	< .05 *
2. Find	ns	< .0001 ****	ns	< .05 *	< .0001 ****	ns	< .001 ***	< .0001 ****	< .05 *	< .05 *
3. Demonstrate	ns	< .001 ***	< .001 ***	ns	< .05 *	< .05 *	ns	ns	< .01 **	< .01 **
4. Determine	< .01 **	ns	< .01 **	< .05 *	ns	ns	ns	ns	ns	ns
5. Know/Known	ns	ns	ns	ns	ns	ns	ns	ns	ns	ns
6. Evidence	ns	ns	ns	ns	ns	ns	< .01 **	ns	ns	< .01 **
7. Prove	< .0001 ****	ns	ns	ns	< .0001 ****	< .0001 ****	< .0001 ****	ns	ns	ns
8. Essential	ns	ns	ns	ns	< .05 *	ns	ns	ns	< .05 *	ns
9. Given	ns	ns	ns	ns	< .05 *	< .01 **	ns	ns	ns	ns
10. Particularly	ns	ns	ns	ns	ns	ns	ns	ns	ns	ns

EF: Earth-to-Formal comparison; EL: Earth-to-Life comparison; EP: Earth-to-Physical comparison; ES: Earth-to-Social comparison; FP: Formal-to-Physical comparison; FS: Formal-to-Social comparison; LP: Life-to-Physical comparison; LS: Life-to-Social comparison; PS: Physical-to-Social comparison.

* *P*<0.05

** *P*<0.01

*** *P*<0.001

**** *P*<0.0001. n = 100 per science discipline. Each n represents one abstract.

**Fig 3:**
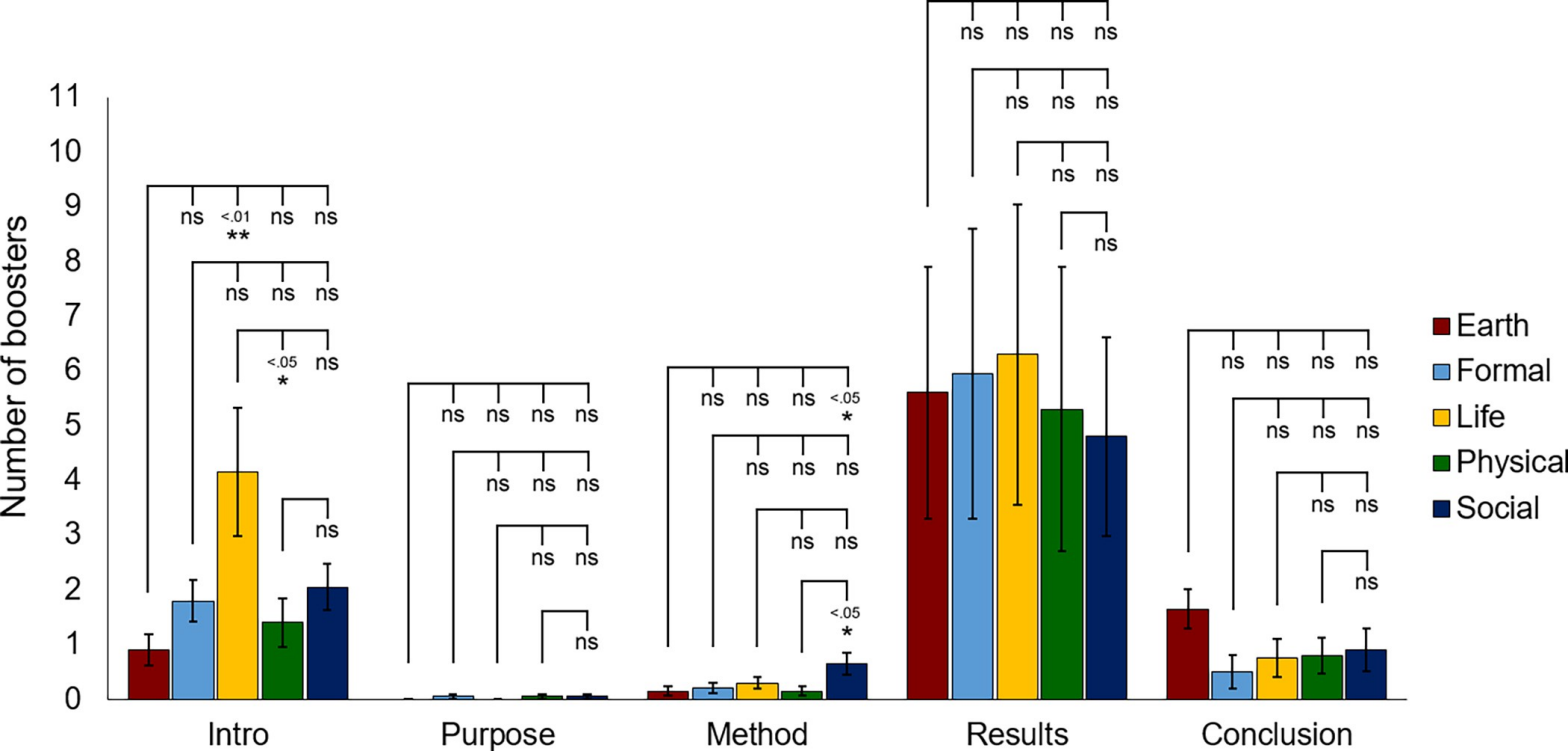
Comparison of boosters’ locations across different science disciplines. Analysis of the number of boosters in different move structure steps in abstracts across various science disciplines. Statistical analysis was performed using ANOVA with post hoc Tukey test. Error bars represent S.E.M. n = 100 per science discipline. Each n represents one abstract.

To gain more insights on the location of these frequently-used boosters within the move structure, we compared the boosters’ locations across different science disciplines. Our data revealed that most boosters were located in the Results move across all science disciplines, followed by those evident in the Introduction and Conclusion moves, which manifested a moderate number of boosters (Fig 3). The Method and Purpose moves contained very few boosters (Fig 3). Interestingly, Life Sciences were found to employ more boosters in the Introduction move compared to Earth and Physical Sciences (Fig 3). Social Science abstracts also incorporated more boosters into the Method move as opposed to Earth and Physical Science abstracts (Fig 3).

### Considerable use of formalization features in earth and life sciences

An appropriate amount of formalization contributes to the demonstration of professionalism, objectivity, and impartiality and could consequently make the abstract worth reading from the perspective of experts of the corresponding discipline [15]. However, over-formalization may make it difficult for non-experts of the discipline and the general public to engage with the text. It has previously been shown that linguistic features at the microdiscoursal level can be quantified and used to measure formalization [17, 26]. Thus, to determine the extent of formalization employed in the scientific article abstracts of different science disciplines (RQ3), we examined the use of linguistic features in various science disciplines. For each of the 500 abstracts, we analyzed the number of words, average word length, number of sentences, minimum sentence length, maximum sentence length, average sentence length, object-verb construction, as well as the use of POS and sentence-initial POS, including the number of nouns, number of noun-beginning sentences, number of pronouns, number of pronoun-beginning sentences and number of WH-pronouns.

**Fig 4:**
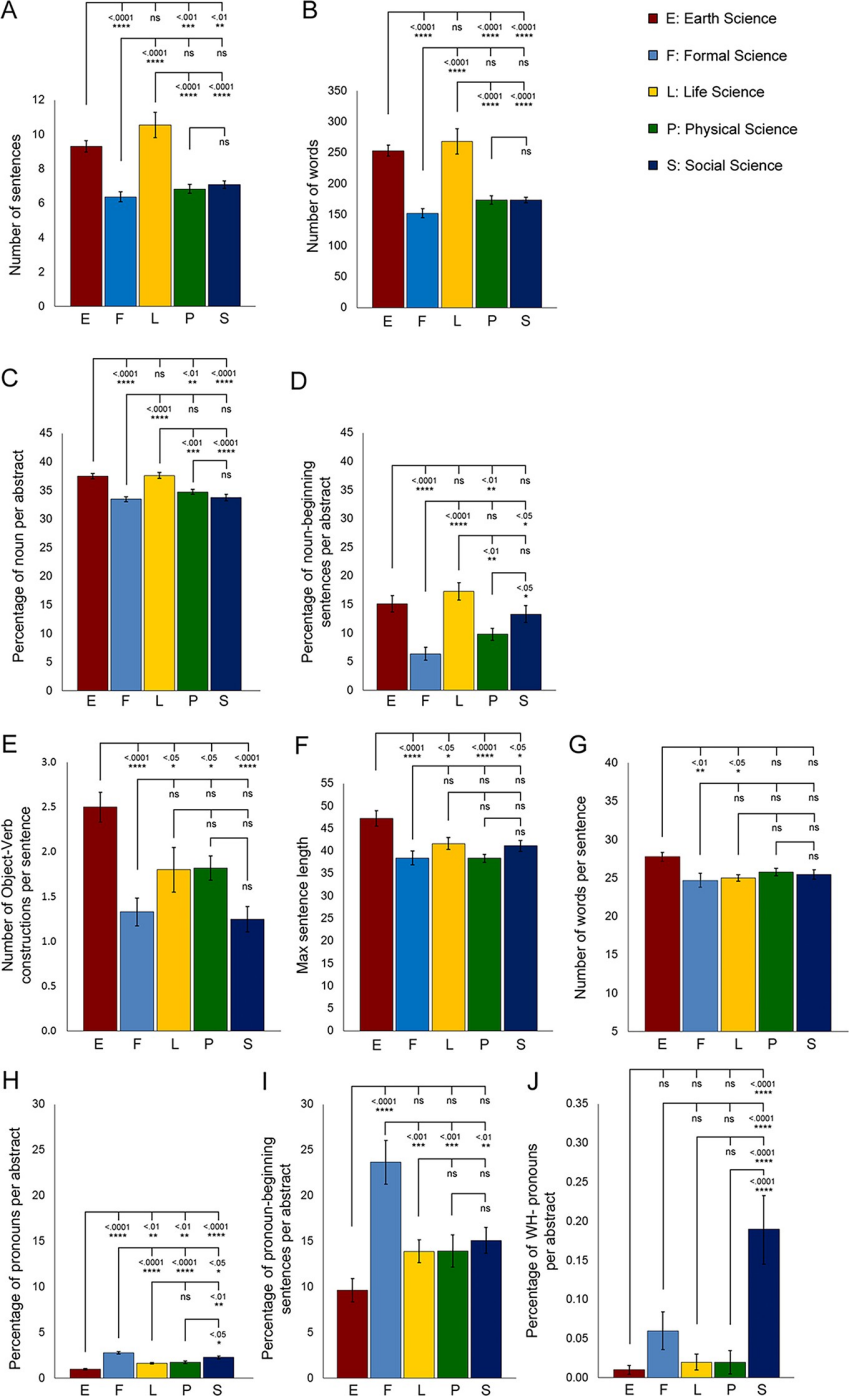
Analysis of formalization features across different science disciplines. (A) Average number of sentences per abstract across five science disciplines. (B) Average number of words per abstract across five disciplines. (C) Percentage of nouns per abstract across five science disciplines. (D) Percentage of noun-beginning sentences per abstract across five science disciplines. (E) Number of object-verb constructions per sentence in the abstracts of five science disciplines. (F) Maximum sentence length in the abstracts of five science disciplines. (G) Number of words per sentence (sentence length) in the abstracts of five science disciplines. (H) Percentage of pronouns per abstract across five science disciplines. (I) Percentage of pronoun-beginning sentences per abstract across five science disciplines. (J) Percentage of WH-pronouns per abstract across five science disciplines. Statistical analysis was performed using ANOVA with post hoc Tukey test. Error bars represent S.E.M. n = 100 per science discipline. Each n represents one abstract.

Our findings show that the work of research journal writers in Earth and Life Sciences displayed a significantly higher mean in the number of sentences (Fig 4A), number of words (Fig 4B) and a more persistent usage of nouns than the other disciplines (Fig 4C). The demonstration of noun-beginning sentences in Earth, Life and Social Science journal abstracts was ascertained as more common than in Formal and Physical Science abstracts (Fig 4D). Moreover, we found a significantly higher number of object-verb constructions and maximum sentence length in Earth Sciences when compared with the other science disciplines (Fig 4E and 4F). Earth Sciences also showed significantly longer average sentence length than those in Formal and Life Sciences (Fig 4G). Overall, these results suggest that formalization is a more frequent characteristic in the abstracts of Earth and Life Sciences than in the other disciplines.

It has been found that the use of pronouns, pronoun-beginning sentences and WH-pronouns is associated with a reduced use of formalization [26]. In our study, the abstracts of Formal and Social Science disciplines demonstrated a higher percentage use of pronouns (Fig 4H). A strong tendency to use pronoun-beginning sentences was also evident in Formal Science abstracts (Fig 4I), whereas Social Science abstracts frequently used WH-pronouns such as What, When, Where, Why and Who (Fig 4J). These results suggest that Formal and Social Science abstracts are more disposed to less formalization than the other science disciplines.

For average word length, despite the fact that a significant difference was witnessed between Formal and Social Sciences, the difference was not salient (S1 Fig). Similarly, no significant difference between groups was observed in the minimum sentence length (S1 Fig).

## Discussion

The abstract of a scientific research article may dictate the success rate of the article being published or a grant application being considered. This is particularly pivotal to researchers of the science community due to the fast-growing competition of publishing and funding acquisition in the past decade [39]. In this study, we provided qualitative and quantitative analyses of the macro-structure, metadiscoursal features such as boosters and microdiscoursal features of scientific research article abstracts from the top 50 ranked journals across multiple science disciplines. Our results have revealed a number of similarities and differences in the features employed by different science disciplines.

### Similarities in the move structure and boosters employed by all five science disciplines

Our data suggest that most science disciplines adopt a 3-step move structure in abstracts (Fig 1A), and all abstracts place a heavy emphasis on the Introduction and Results moves, with particular attention to the Results move (Fig 1B) validating the findings obtained from a computational analysis in a previous study [31]. The Introduction move not only provides background information about the topic but more importantly, actualizes the rationalization strategies of cause and problem [17], and highlights the significance or value of the topic to readers [4]. The Results move is important since it manifests the rationalization strategies of effects and solutions [17]. Original findings are highlighted in the Results move as a means of informing readers that the article is worth reading. Consistent with these findings, boosters are most frequently found in the Introduction and Results moves in abstracts, with the highest frequency observed in the Results move across all disciplines (Fig 3). In the Introduction move, boosters highlight the novelty, impact and value of the research, whereas in the Results move, boosters demonstrate the strength of the association between the data and claims/findings [4]. Since originality and new findings are of crucial importance in academic advancement and obtaining funding for research, the Introduction and Results moves are, as expected, the most frequently observed, and boosters employed in these moves serve to strengthen the value of the abstracts and persuade readers to continue reading the scientific articles.

### Interdisciplinary variations in the move structure, use of boosters and formalization features

The uncovering of noticeable variations in terms of how macro-structure, metadiscoursal elements and microdiscoursal features are used in abstracts of each of the five science disciplines marks a key contribution as offered in our study. Notably, in Earth Science journal articles, the booster “determine” is much more frequently employed than in those of Formal, Physical and Social Sciences (Tables 4 and 5). Earth Science writers also exhibit more formalization features with a higher number of sentences and words, nouns, noun-beginning sentences, object-verb constructions, maximum sentence length and average sentence length (Fig 4A to 4G). High levels of formalization can reduce ambiguity and convey impartiality [15]. This tends to make the research more objective and credible to experts of this discipline, but at the same time, less appealing to non-experts and policy-makers.

In line with the other science disciplines, Life Sciences have a high adoption rate of the Results move in abstracts (100/100 abstracts) (Fig 1B). However, unlike the other disciplines, Life Sciences highly favor employing the Introduction and Conclusion moves in abstracts (Fig 1B). The Conclusion move is more frequently observed in Life Science abstracts because this discipline can sometimes be interpretive and inferential, whereby theories are proposed and then tested, and informing peers of the value of the research is often required [4]. Life Sciences frequently adopt the I-R-C initiate move structure (Tables 1 and 2), whereby the need for the research, results, generalizations of findings, and the significance and value of research are all highlighted, providing more transparency and accessibility to scientific knowledge.

One noteworthy finding is that the Life Science discipline often includes additional moves such as funding schemes, theme issues and journal information (Fig 2), indicating that elaboration in writing is often expected in this discipline whose intended audience may include non-experts and policy-makers. This finding sheds light on abstracts as a genre with respect to revealing a distinct move structure for Life Science abstracts. Scientists from this discipline also apparently employ more metadiscoursal features such as boosters for persuasion than other science disciplines (Table 3). In particular, “show”, “find” and “demonstrate” are frequently used in these abstracts (Tables 4 and 5). All disciplines employ the use of boosters mainly in the Results move of abstracts (Fig 3). But interestingly, the Life Science discipline frequently uses boosters in the Introduction move as well (Fig 3). Boosters enable scientists to emphasize the novelty of their findings and increase solidarity with their readers [14, 17, 23]. It is likely that the stiff competition for research grants in Life Sciences drives scientists in this discipline to employ more boosters in their abstracts so as to strengthen their findings and value of the study. A recently published study in PNAS has confirmed that the average age for an investigator receiving his/her R01 or equivalent grants in the United States rose from less than 38 years old in 1980 to more than 45 years old in 2013 owing to the intensity of vying for funding in biomedical science research [40].

Similar to writers for Earth Science research journals, those working in Life Sciences exhibit a high degree of formalization in abstracts, adopting a distinct writing style whereby they prefer to use long sentences and noun-beginning sentences (Fig 4A to 4D). Even though there are general guidelines on science writing in that it should be precise, clear and concise, and that scientists should avoid using an impersonal style but instead use first person pronouns [9, 41], a reasonable amount of formalization is required to demonstrate objectivity and avoid misinterpretation of information [15]. However, the suggestion that excessive use of formalization in abstracts might reduce conciseness is a point worthy of consideration.

Compared with abstracts from the other disciplines which mostly adopt the 3-step move structure, those abstracts in the field of Formal Sciences have a tendency to employ fewer steps (Fig 1). The majority of these abstracts consist of the simple I-R-only and R-only move structures (Tables 1 and 2). It is possible that Formal Science abstracts are primarily intended for experts in that field, assuming that they have specialized knowledge of the way the study was conducted and even the background knowledge. Experts can access their shared understanding of the discipline to determine the significance of the study and the procedures employed. Concerning the use of boosters, abstracts in the Formal Sciences frequently employ “prove” and “given” (Table 3, Fig 3A and 3B). Since the discipline of Formal Sciences has a stronger orientation to fixed notions of knowledge, claims or findings in this discipline would likely rely more on empirical data resulting from universally accepted principles of inquiry and methodological rigor rather than on the unique or novel ideas of scientists [42]. Therefore, stronger boosters such as “prove” and “given” are much more frequently employed. The Formal Science discipline incorporates a high number of pronouns and pronoun-beginning sentences into their abstracts (Fig 4H and 4I), indicating a comparatively lower level of formalization. Previous studies have found that first person pronouns help to establish the authority of the writer [15], which may thus assist scientists of this discipline in presenting research to their peers in their academic community.

The Social Science discipline has the highest percentage of abstracts with the Methods move among all five science disciplines (Fig 1B) and it employs more boosters in the Methods move than Earth and Physical Sciences (Fig 3). The vast complexity and broad nature of Social Sciences (e.g. human behavior) may lead scientists to explain the method in more detail and qualify their knowledge claims by using words such as “evidence.” As found in a previous study, social sciences often require thorough interpretation of qualitative and quantitative analyses in constructing and constituting knowledge [43]. Therefore, abstract writing in social sciences may involve detailed elaboration on how the research is conducted and data is interpreted.

Similar to Formal Scientists, Social Scientists employ a high percentage of pronouns (Fig 4H) and exclusively exhibit the highest percentage of WH-pronouns when compared with the other four disciplines (Fig 4J), suggesting that this discipline employs a low level of formalization. It is likely that the reduced use of formalization facilitates understanding of the research study by non-experts and policy-makers so as to obtain support for findings [15]. This discipline is based less on empiricism when compared with hard sciences, and the use of pronouns can establish the authority of the researcher, and hence engage readers in arguments and support for findings.

Compared to the other four disciplines, the Physical Sciences have the highest number of abstracts consisting of all 5 moves: I-P-M-R-C (Fig 1A). It seems that scientists in this discipline accommodate their academic peers and non-experts by highlighting all details such as the area to be covered, the aim, how the study was done, the key findings and the value of the study in the abstract. Writing in the Physical Science discipline also has more reverse sequences in the move structure than that of Life Sciences (Fig 2), which warrants further investigation. Although the use of boosters in the texts of the Physical Science discipline is markedly limited in comparative terms (Table 3), the word “demonstrate” (Tables 4 and 5) which focuses on the strength of the relationship between the data obtained and the claims made is customary [4].

### Observing cross-disciplinary conventions

By analyzing the genre of research article abstracts within the science community, we discovered a significant number of variations in the macro-structure, metadiscoursal and microdiscoursal features exhibited in different science disciplines. Each science discipline possesses its own unique discipline-specific conventions in the use of the move structure for rationalization, the use of boosters for persuasion and linguistic features for formalization. As cross-disciplinary collaborations in the science community are becoming increasingly common, interdisciplinary communication is becoming an emerging theme of interest for scientists. Journal editors and panel members of funding committees often include scientists from different disciplines or sometimes even non-scientists. Therefore, it is suggested that scientists observe disciplinary conventions when writing abstracts to present their research studies to scientists from other disciplines more effectively. Understanding and following these distinct cross-disciplinary conventions may help scientists to make their abstracts worth reading, and ultimately increase the success rate of publications and grant acquisitions. Moreover, scientists should avoid over-formalization to increase the readability of their abstracts if the target audience includes non-experts or the general public.

### Future research

Further research could measure how language use contributes to goals such as obtaining a grant, support for a policy or the acceptance of a publication, which scientists aim to achieve. Interviews could be conducted with scientists from a few disciplines to corroborate the results from our study. Experimental studies could be designed to examine whether different science disciplines indeed have their distinct move structure as manifested in rationalization strategies and employ metadiscoursal features such as boosters coupled with an avoidance of excessive formalization.

## Conclusion

In summary, by analyzing the abstracts of scientific research articles sampled from top journals across five science disciplines, our study reveals interdisciplinary variations in the move structure, use of boosters and linguistics features exhibited in abstracts. In terms of academic research, our work contributes to cross-disciplinary investigation of the macro-structure, metadiscoursal and microdiscoursal features from multiple science disciplines. Specifically, we have found that abstracts from each particular science discipline have their distinct move structure, use of boosters and linguistics features. This has implications for our understanding of how these rhetorical choices in abstracts are made by scientists in each distinct discipline in the contemporary institutional context in which competition for publications and grants has exacerbated.

In terms of application, our results provide insights for scientists involved in interdisciplinary collaborations who are interested in publishing or applying for grants cross-disciplinarily. By observing distinct disciplinary conventions, scientists can align the abstracts of their studies with the expectations of the targeted audience. In addition, scientists should be cautious when their targeted audience includes non-experts, such as policy-makers and the general public. Proper use of rationalization strategies, metadiscoursal features for persuasion and avoidance of over-formalization are recommended to ensure that research is made more accessible to non-experts while maintaining credibility.

## Supporting information

S1 Fig Analysis of formalization features (average word length and minimum sentence length) across different science disciplines. (A) Average word length per abstract across five disciplines. (B) Minimum sentence length per abstract across five disciplines. Statistical analysis was performed using ANOVA with post hoc Tukey test. Error bars represent S.E.M. n = 100 per science discipline. Each n represents one abstract.
(TIF)

S1 File List of Top 50 journals. 50 journals of the highest-impact factor from the sub-disciplines of the five major science disciplines (Earth, Formal, Life, Physical and Social Sciences) were identified from InCites Journal Citation Report. To avoid content and structural bias, journals that had a standardized abstract format and/or focused solely on publishing review articles were not selected even if they had a high-impact factor. Two sub-disciplines were selected under each of the five science disciplines. One category was selected from the InCites Journal Citation Reports to represent one sub-discipline, if appropriate. Two or more categories were included in a sub-discipline if a single category was not available.
(XLSX)
