# Supplementary figures and images for: A discourse analysis of the macro-structure, metadiscoursal and microdiscoursal features in the abstracts of research articles across multiple science disciplines
Source: PLoS One. 2018 Oct 12;13(10):e0205417. doi: 10.1371/journal.pone.0205417 (PMC6185845; doi:10.1371/journal.pone.0205417)

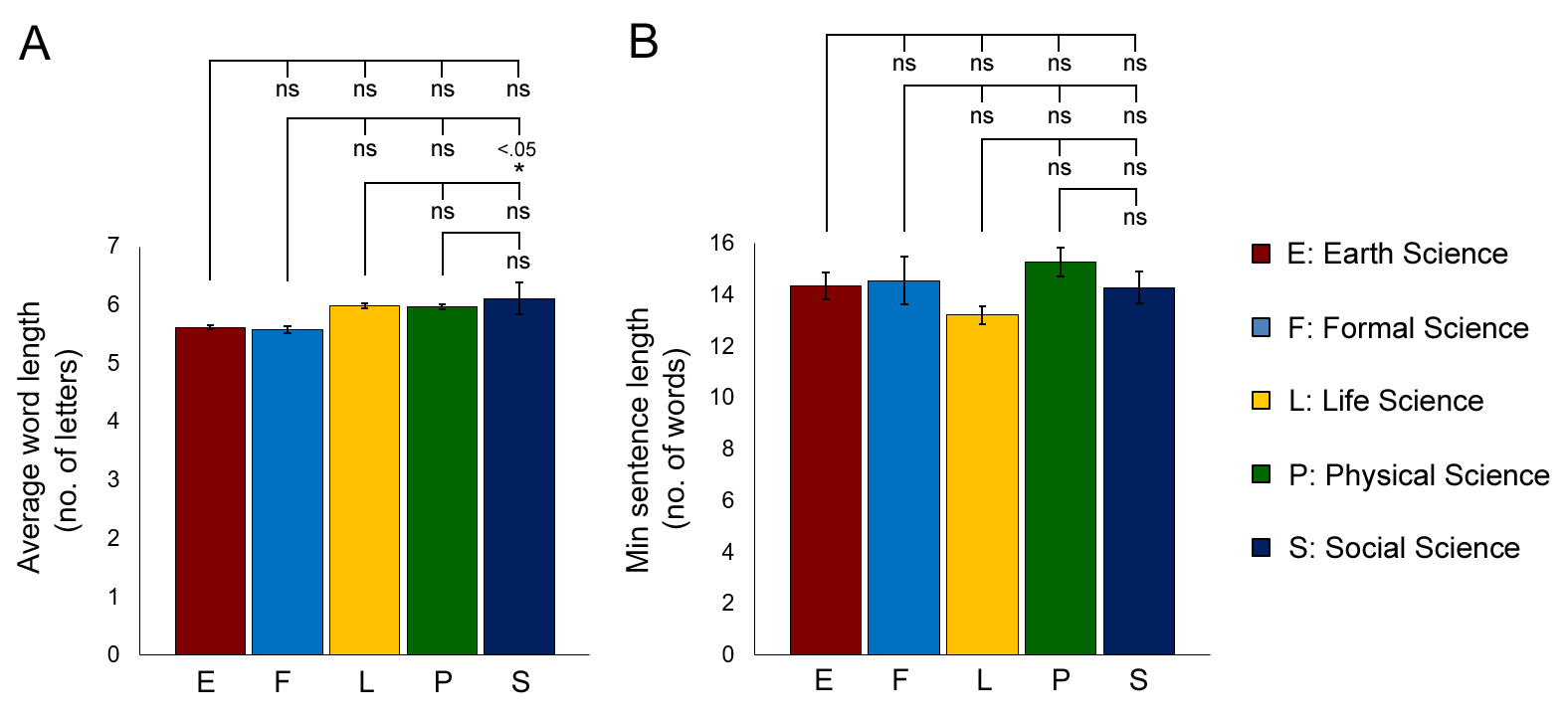

Supplement: S1 Fig — (A) Average word length per abstract across five disciplines. (B) Minimum sentence length per abstract across five disciplines. Statistical analysis was performed using ANOVA with post hoc Tukey test. Error bars represent S.E.M. n = 100 per science discipline. Each n represents one abstract. (TIF) [file pone.0205417.s001.tif]
